# E-learning for medical imaging specialists: introducing blended learning in a nuclear medicine specialist course

**DOI:** 10.1177/2058460117720858

**Published:** 2017-07-25

**Authors:** Torjan Haslerud, Andreas Julius Tulipan, Robert M Gray, Martin Biermann

**Affiliations:** 1 Nuclear Medicine and PET Center, Department of Radiology, Haukeland University Hospital, Bergen, Norway; 2 Department of Nuclear Medicine, Oslo University Hospital, Oslo, Norway; 3 Department of Education, University of Bergen, Bergen, Norway; 4 Department of Clinical Medicine, University of Bergen, Bergen, Norway

**Keywords:** Education, radionuclide studies, computed tomography (CT), single-photon emission computed tomography (SPECT), positron emission tomography (PET)

## Abstract

**Background:**

While e-learning has become an important tool in teaching medical students, the training of specialists in medical imaging is still dominated by lecture-based courses.

**Purpose:**

To assess the potential of e-learning in specialist education in medical imaging.

**Material and Methods:**

An existing lecture-based five-day course in Clinical Nuclear Medicine (NM) was enhanced by e-learning resources and activities, including practical exercises. An anonymized survey was conducted after participants had completed and passed the multiple choice electronic course examination.

**Results:**

Twelve out of 15 course participants (80%) responded. Overall satisfaction with the new course format was high, but 25% of the respondents wanted more interactive elements such as discussions and practical exercises. The importance of lecture handouts and supplementary online material such as selected original articles and professional guidelines was affirmed by all the respondents (92% fully, 8% partially), while 75% fully and 25% partially agreed that the lectures had been interesting and relevant.

**Conclusion:**

E-learning represents a hitherto unrealized potential in the education of medical specialists. It may expedite training of medical specialists while at the same time containing costs.

## Introduction

Since the late 1990s, e-learning has gradually been established as an important tool in higher education, including medical studies (1). Incentives for e-learning in higher education have included cost savings for academic institutions that were short of academic staff and the drive to barrier-free education irrespective of constraints in time and space. A more recent trend has been the introduction of blended learning in which face-to-face time (F2F) in a classroom is enhanced by online learning, particularly in the form of activities and resources (2). A recent large meta-analysis by the United States Department of Education concluded that blended learning was significantly more effective than fully F2F or online courses (3).

It is therefore surprising that the professional training of medical specialists is still largely based on lecture-based courses even though the shortcomings of a pure lecture format have been amply documented (4–6). In Scandinavia, a specialist in radiology or nuclear medicine (NM) will typically attend two months of courses in the course of her five-year-long professional training (7). Given the time and resources spent on courses for medical specialists, it is very important that course design and teaching strategies promote optimum learning outcomes.

To assess the potential of e-learning in specialist education in medical imaging, we extended the customary lecture format of the obligatory course in clinical NM with e-learning elements in an online learning management system (LMS) (8). We evaluated the resulting blended course format in an anonymized user survey conducted after completion of the course.

## Material and Methods

The course “Clinical Nuclear Medicine” was held at our institution during 7–11 November 2016 as Norwegian Physicians’ Association (Dnlf) course 31043. The course, comprising 30 h of lecture-based teaching in line with current regulations, was enhanced using e-learning material on our new national teaching platform for NM, which includes a LMS exposed to the Internet and DICOM server with anonymized teaching cases inside the Norwegian health network (8).

The online course material included handouts of all lectures in Portable Document Format (PDF), which in previous courses had been distributed on paper, as well as supplementary materials such as professional guidelines, selected open access articles, and institutional procedures for specialized examinations. The course was further enhanced by optional practical exercises. Course participants, in groups of two, used the clinical imaging workstations at our center to review selected anonymized cases in the national teaching database in three sessions of one hour each. The candidates were presented clinical referral information and asked to write an imaging report inside the LMS as previously described (8). The course was concluded by an online examination in the LMS one week after the classroom sessions. The examination comprised 136 multiple choice questions covering all topics presented in the lectures. The pass criterion was 80% correct answers. Participants were allowed re-take the examination multiple times after a refractory period of 1 h. In addition, they were encouraged to discuss difficult questions in dedicated discussion forums between attempts.

**Fig. 1.**
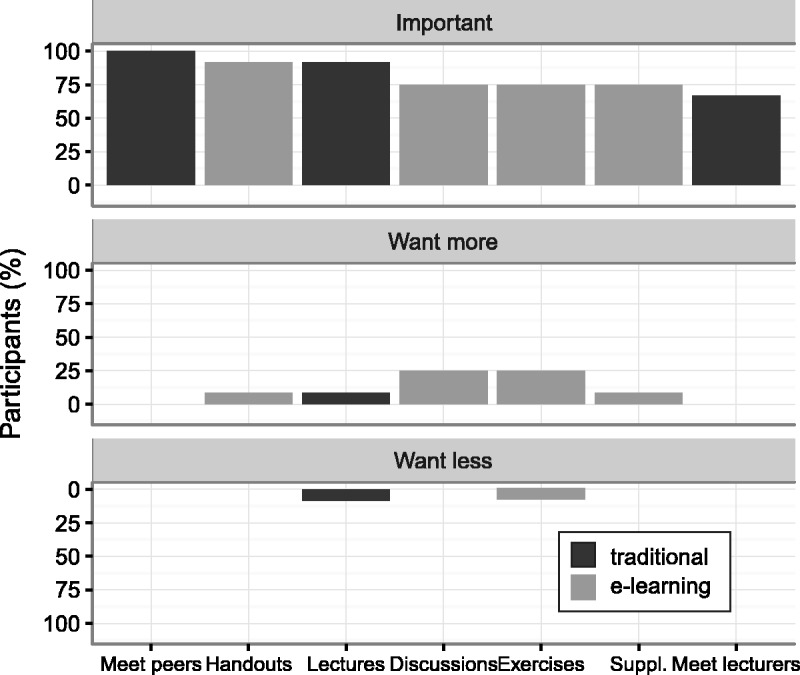
User feedback on the importance of learning elements in specialist education. The plot shows the proportion of participants who affirmed that a given item was “important,” and who wanted more and less of a given item in specialist education. Traditional learning elements are plotted in black, e-learning elements in gray. Items are ranked in order of their perceived importance. No participants chose the category “others,” which is omitted from the plot to enhance clarity. Suppl. = supplementary materials such as professional guidelines and original articles.

After the course examination was completed, an anonymized survey comprising 20 questions was presented to the students via the Moodle “Feedback” activity (Fig. 1; Supplementary Materials 1).

The survey and the consent form had been approved by the data protection officer at Haukeland University Hospital. Analysis and plotting were performed as previously described (9).

## Results

The course was attended by 15 participants. These included all physicians currently completing their course education in NM specialist training in Norway as well as two physicians in specialist training in radiology. All participants passed the course examination after an average 1.7 attempts (range = 1–6). Twelve of 15 course participants (80%) took part in the survey.

As shown in Fig. 1, overall satisfaction with the blended course format was high. However, 25% of the course participants wanted more interactive elements in the form of discussions and exercises. Ninety-two percent fully agreed and 8% partially agreed that the extra learning material put out on the LMS was useful, while only 75% fully agreed and 25% partially agreed that the course lectures were interesting and relevant. Fifty percent fully agreed and 33% partially agreed that the discussion forums and the practical exercises were useful. Lectures and handouts were rated as important by 92%, while all participants unanimously agreed on the importance of personal contact with the other participants (Fig. 1). The most frequently cited benefit from e-learning in 7/10 free-text responses was easy access to handouts and supplementary materials, both before and after the course, while two participants mentioned the ability to communicate and discuss with other participants. There was unanimous agreement (75% full, 25% partial) that the LMS was easy to use.

The most criticized aspect of the course was the course examination, which was deemed as too difficult and too time-consuming in relation to the lectured material by half the respondents even though two-thirds of the respondents agreed that the questions had been relevant (25% fully, 42% partially). The detailed survey results are available as Supplementary Materials 1.

## Discussion

We report on the first blended course in Norwegian medical specialist education. Overall, the participants who took part in the survey (80%) were highly satisfied with the course, including its electronic enhancements.

One of the reasons blended courses are significantly more effective than fully F2F or online courses is that students are asked to do more work through active engagement with the material than in traditional lecture-based courses (3).

Despite high satisfaction with the blended course format, personal contact between the course participants was still the most often quoted important ingredient in the learning situation, cited by 100% of the responders. This finding in our study implies that F2F time is needed and should not be fully replaced by an online learning environment.

The most criticized aspect of the course was the examination, which was deemed by half the participants as too difficult and time-consuming. One-third of the participants would have preferred more time for discussion and for the practical exercises. The organizers of the course were aware of these potential issues and are about to launch two new 30-h e-courses, one on organ imaging and one on cancer imaging, the first already approved by the Dnlf (8).

Improving the efficiency and effectiveness of specialist training in medical imaging is of vital importance given the impending shortage of specialists (10–12). In the Nordic countries, much of the education of specialists takes place in courses. In order to become a specialist in radiology or NM in Norway, a candidate must attend some 250 h of mandatory courses, amounting to a total of approximately two months in the course of a five-year education (13). The Danish and Swedish systems for specialist training are based on very similar requirements (7).

Nearly all basic courses in specialist education are lecture-based, even though research into teaching modes in higher education has clearly shown that lectures are the least efficient mode of transmission of information (4–6). Only very few courses are organized as workshops, and these are usually at a high level of subspecialization for a small number of participants due to the difficulty of procuring patients or suitable phantoms for hands-on teaching of intellectual and manual skills.

The impending reform of specialist training in Norway (12) increases the need for efficiency in medical training. In line with modern didactics, some 60–100 teaching goals have been defined for each specialty. These are tied to the completion of defined learning activities, such as performing a certain number of procedures, as well as attending a prescribed number of courses. Delivering lecture-based courses at the required frequency of once or twice a year will be impossible to achieve for specialties or subspecialties such as NM or pediatric radiology that have few accredited specialists. We therefore predict that e-learning will play a major role under the new system.

Image-based disciplines such as radiology and NM can be taught economically using a blended format. Our new national e-learning platform (8) can easily be extended and adapted to other imaging disciplines such as pathology (14). The medical imaging database facilitates instruction also to be based on rare teaching materials. This is highly relevant in NM, where many important protocols are not available at every institution (15), and in specialized fields of radiology, such as pediatric imaging. The proposed e-course format is in several ways similar to problem-based learning (PBL), including an emphasis on problem solving, collaboration, and real-world applications (16), and with some adjustment can be easily adapted to include elements of both PBL and team-based learning (17). Problem-based tutorials have also been successfully employed in radiology education for medical students (18).

Our study has several limitations. First, our blended course was limited to only a small number or participants. This limitation was unavoidable given the small number of NM trainees in Norway. However, we are extending the blended course architecture to a number of larger courses in other related disciplines (https://nukit.ihelse.net/moodle). Second, the survey primarily assessed student ratings rather than teaching outcomes. Correlating student ratings with teaching outcomes would have needed a larger sample size. However, student ratings of teaching effectiveness have been consistently shown to be positively correlated with higher and more lasting student achievement (19) while student dissatisfaction impedes significant learning (20). Third, the largest part of our course was still lecture-based. This was a requirement so that the present course maintained approval by the Dnlf. A new e-course on NM organ imaging is in preparation (8). Lastly, the survey did not elucidate the different levels of professional experience of the course participants. Including demographic questions would have impacted the anonymized design of our survey.

In conclusion, blended learning based on the combination of classroom instruction and online teaching is an important emerging mode of instruction also in specialist education. When combined with meaningful online activities such as reporting on imaging studies in diagnostic format, it can enhance learning while freeing classroom time for discussion and critical reflection.

## Supplementary Material

Supplementary material
