# Supplementary material for: E-learning for medical imaging specialists: introducing blended learning in a nuclear medicine specialist course
Source: Acta Radiol Open. 2017 Jul 25;6(7):2058460117720858. doi: 10.1177/2058460117720858 (PMC5533268; doi:10.1177/2058460117720858)
Supplement: Supplementary material [file E_Learning_Supplementary_Material_720858.pdf]

Supplementary Materials 1: User survey

The user survey was conducted in Norwegian using the Moodle "Feedback" activity under Moodle 2.9. The following is the translated output of the Moodle "Feedback" analysis function. To produce graphs with English labels, the entire survey was imported into a development system running Moodle 3.2 under Oracle Linux 7.3 and each question updated with choices translated into English.

The labels on the x-axes in the plots represent number of respondents. The plots are normalized to the longest bar in the plot, not to the total number of respondents. This is a Moodle default that cannot be overridden. Each bullet in the answers to the free text questions represents the feedback of one anonymous respondent.

The authors' comments on the survey including supplementary detailed are typed in *italics*.

Submitted answers: 12

Questions: 20

1. The lectures were interesting and relevant.

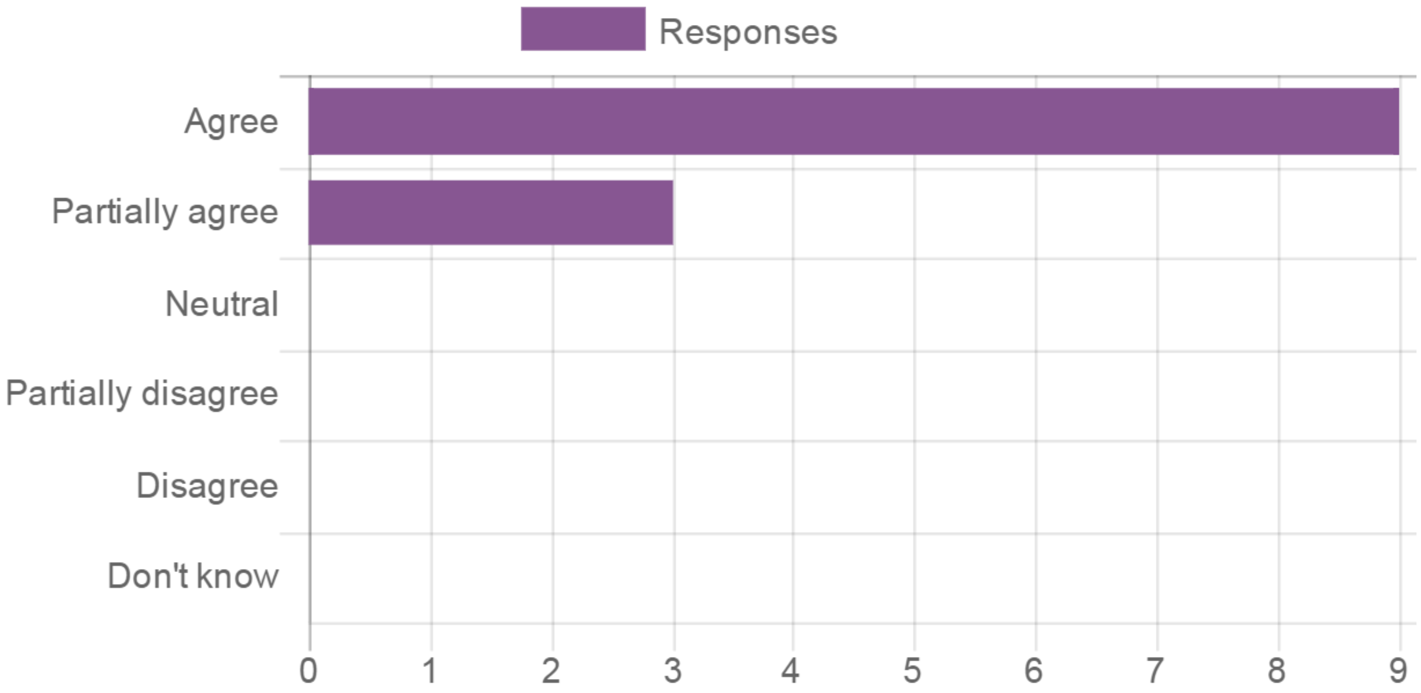

2. The lecturers were motivated.

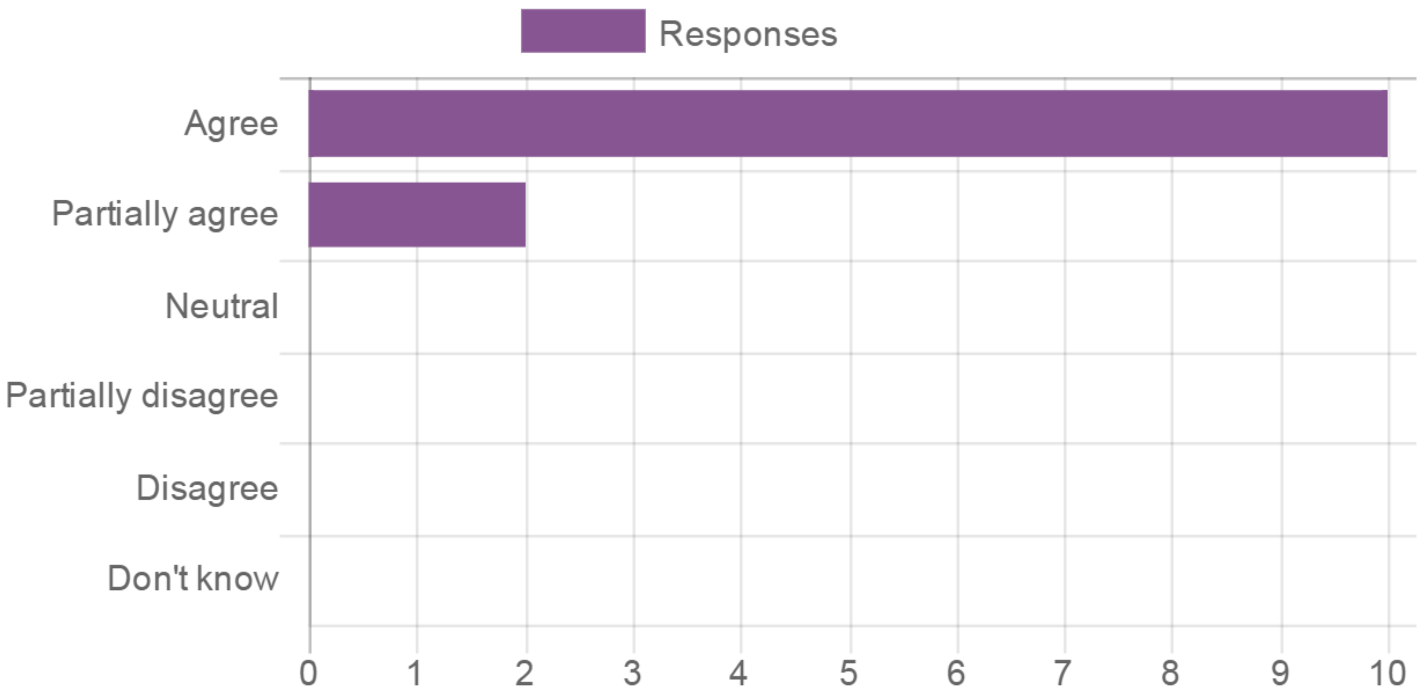

3. The meeting facilities were good.

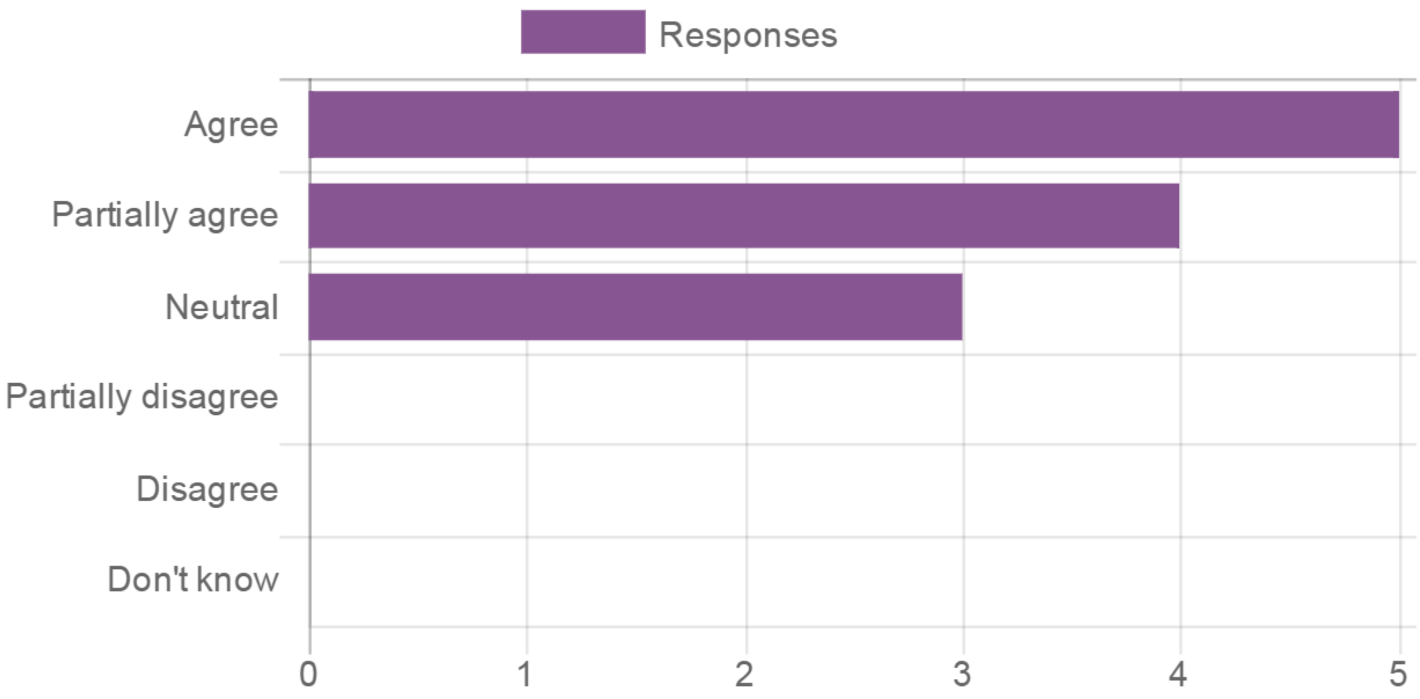

4. The course examination was…

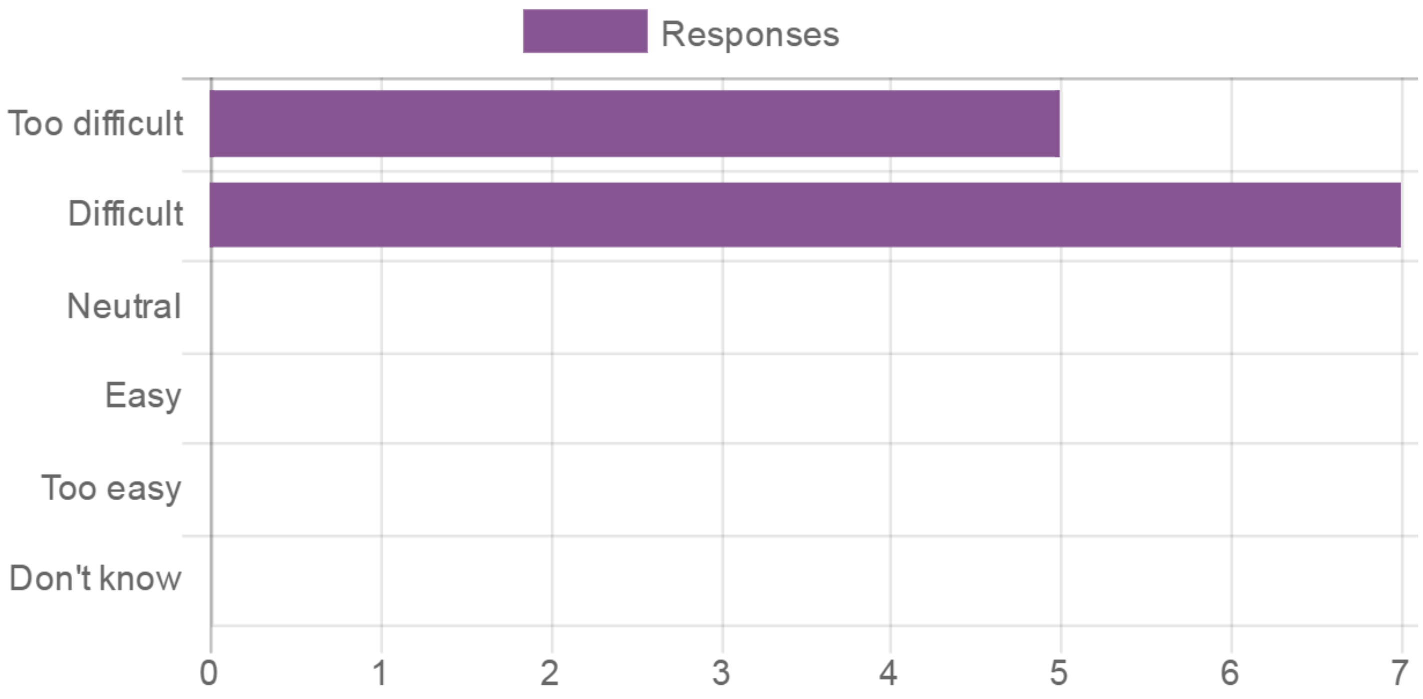

5. The questions in the course examination were relevant.

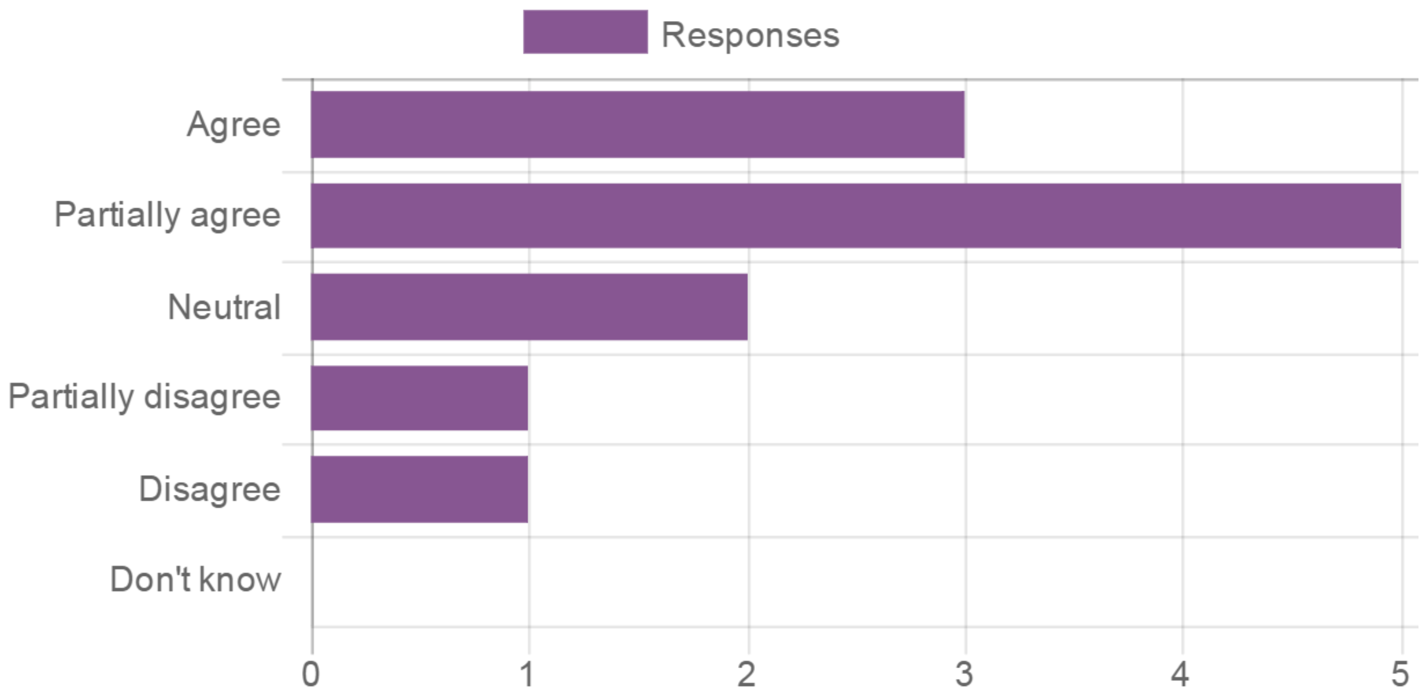

6. Lectures are the best method to impart knowledge.

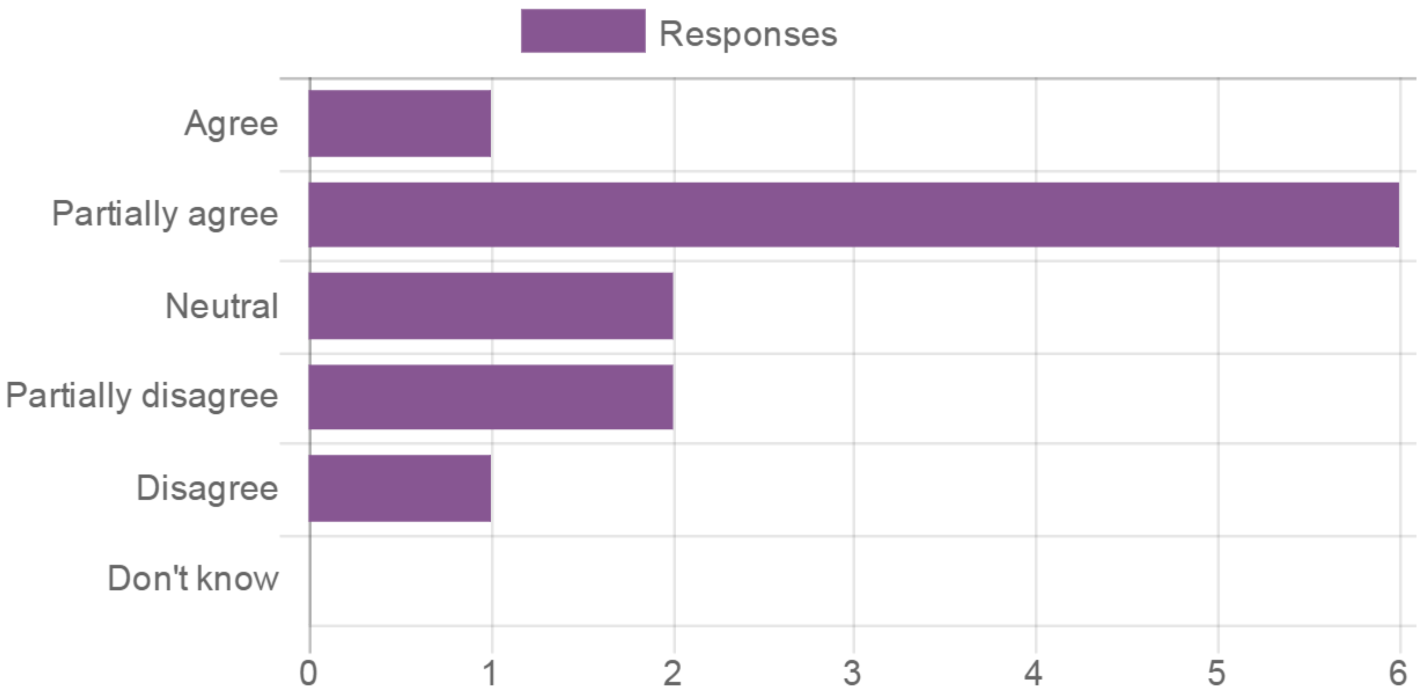

7. Handouts and other materials (articles, guidelines) in addition to the lectures have been useful.

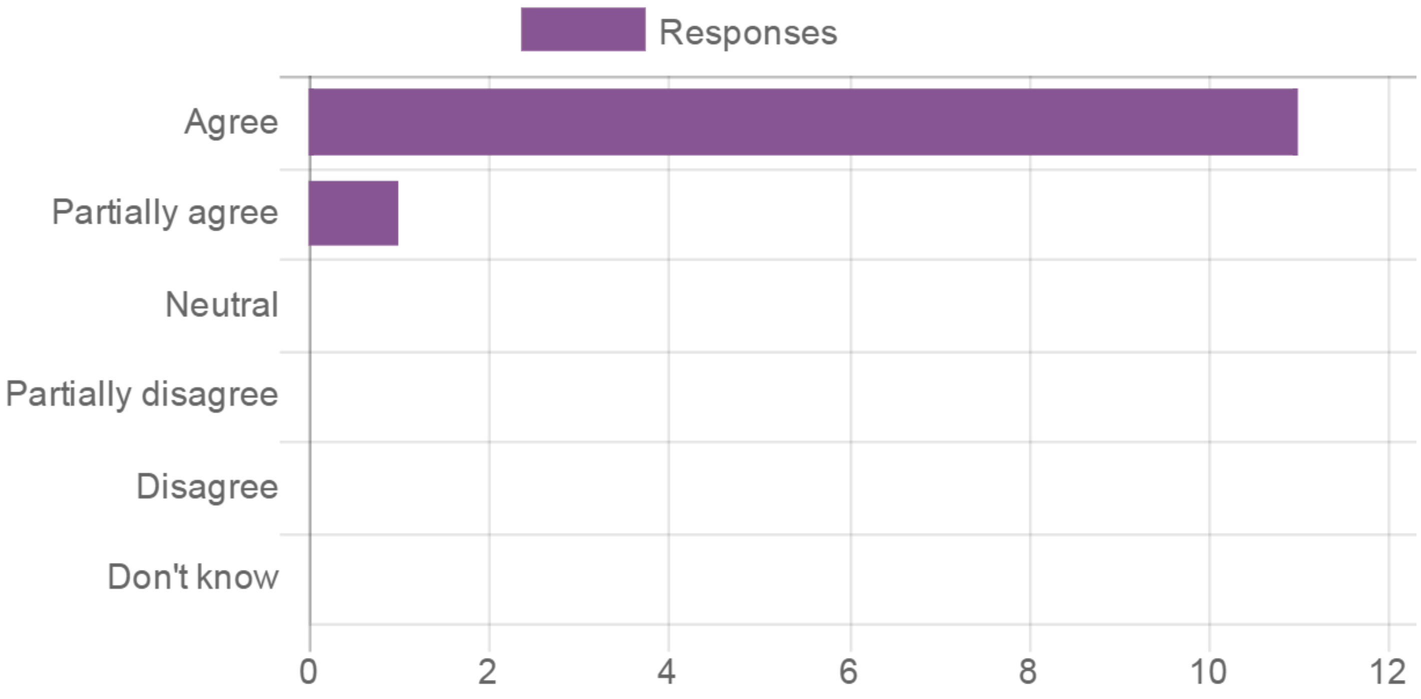

8. Practical exercises with patient cases have been useful.

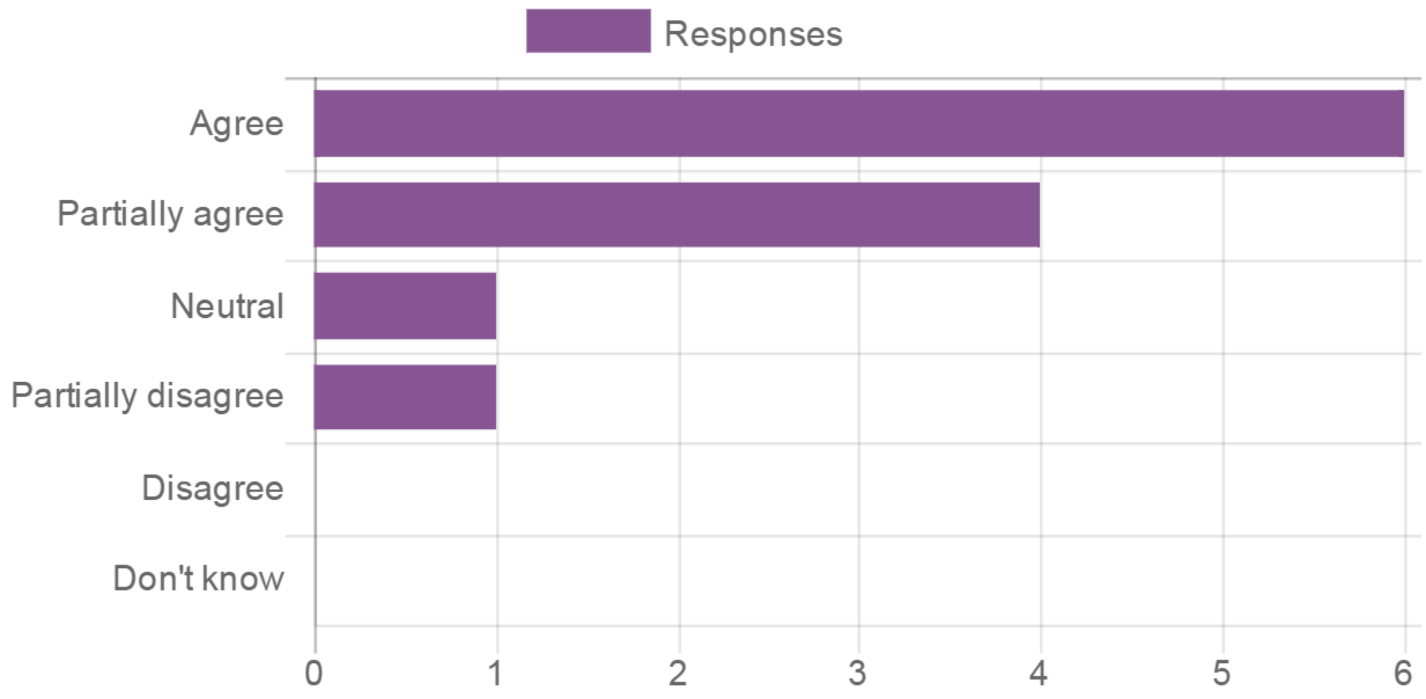

9. Discussion forums are a useful tool.

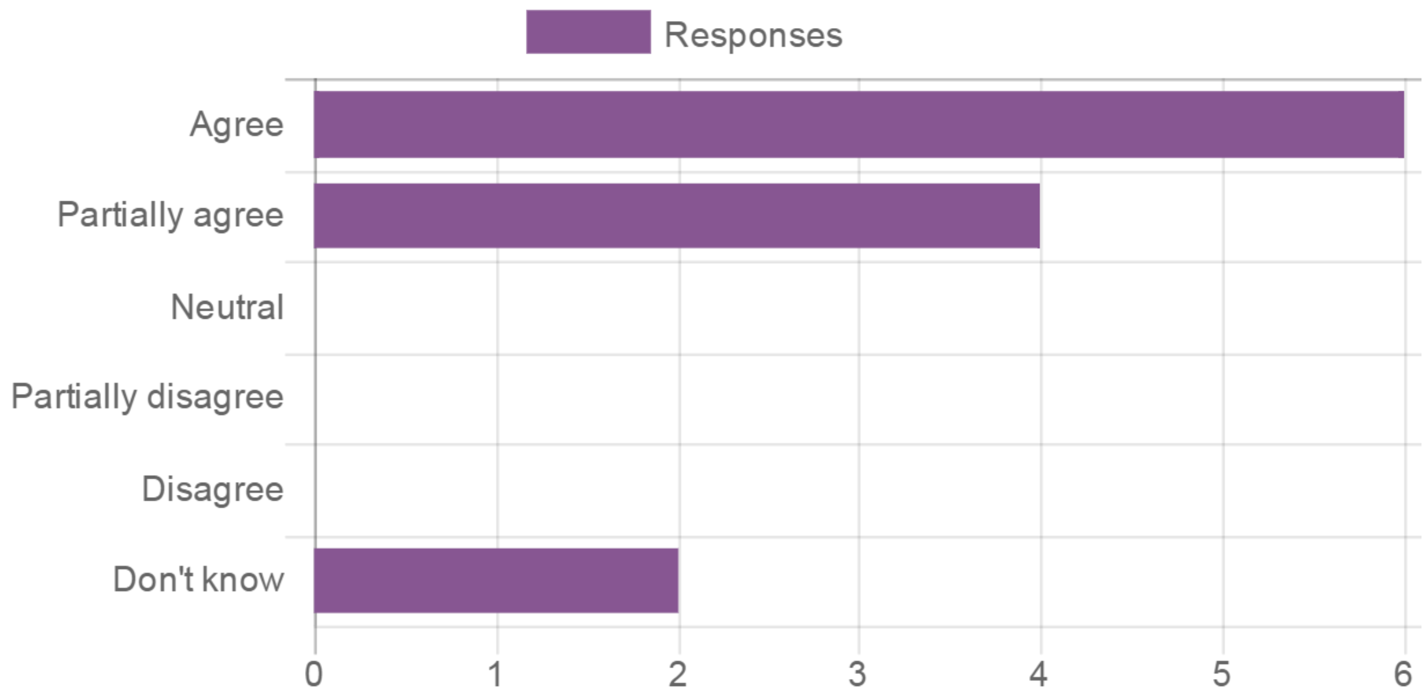

10. The Moodle e-learning platform was easy to use.

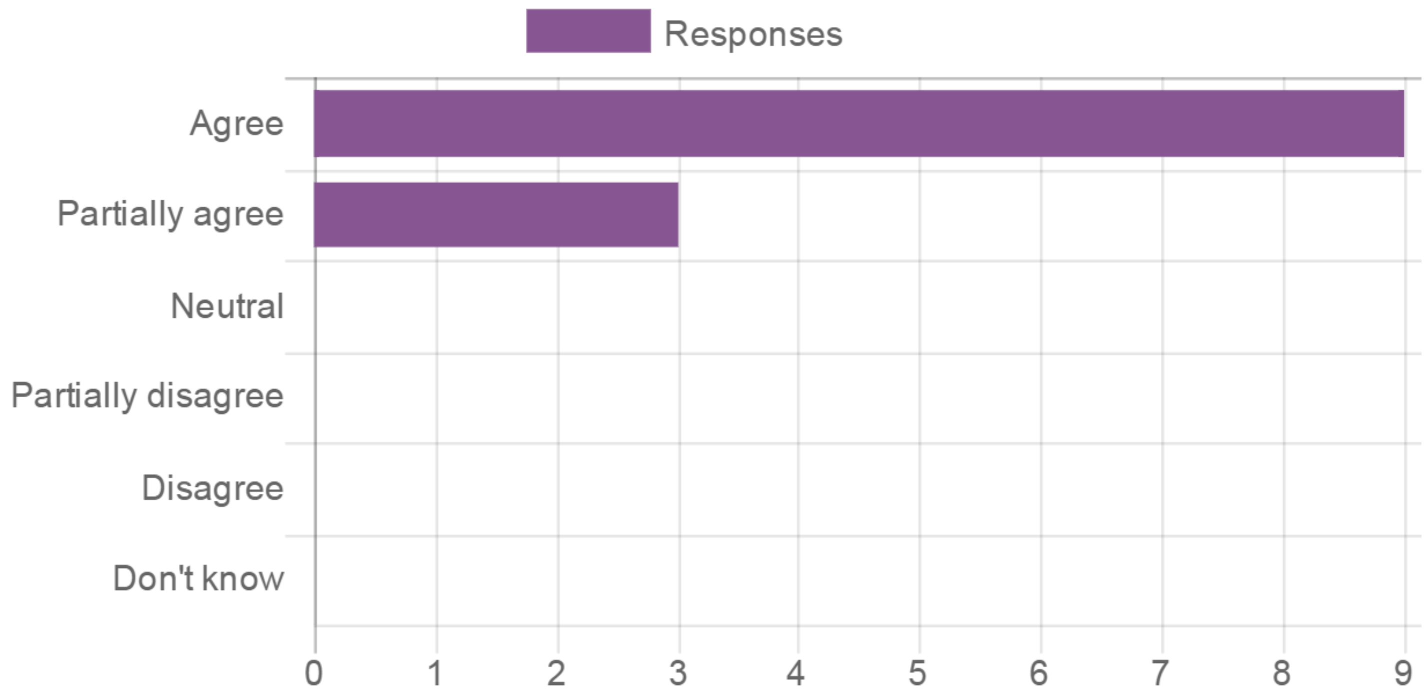

11. What has been the greatest benefit of adding e-learning (= Moodle) to the course? Please give a few key words or tell if you believe that e-learning has been irrelevant or distracting.

- Good overview over lectures and lecture notes.
- It is fantastic that one can go back to the lectures after the course. I learn most when I can sit for myself in peace and combine different sources (books, *UpToDate*, and for example these lectures).
- Opportunity to continue to work with the lectures after completion of the course.
- Very straightforward with handouts and guidelines in one place that you have access to well after the course. Useful with discussion forum (even though it was not yet widely used now).
  There was some little time for practical exercises, could possibly have arranged earlier on each day? Hard to get the benefit of practical tasks if none in the group had experience with Oasis. Took a long time to write the answers - one suggestion might be to read background info, look at the examination, discuss and then respond by means of multiple choice.
- All course materials are in one place. Easy to navigate. I can download the lectures to my own PC.
- Provides a good overview, makes it easy to communicate with other participants, easy to go back and find the information, don’t have to print out handouts on paper.
- Can use info also at work after the course up to 1 year.

12. The following points are important to me in a course in specialist education. Mark all items that you think are important.

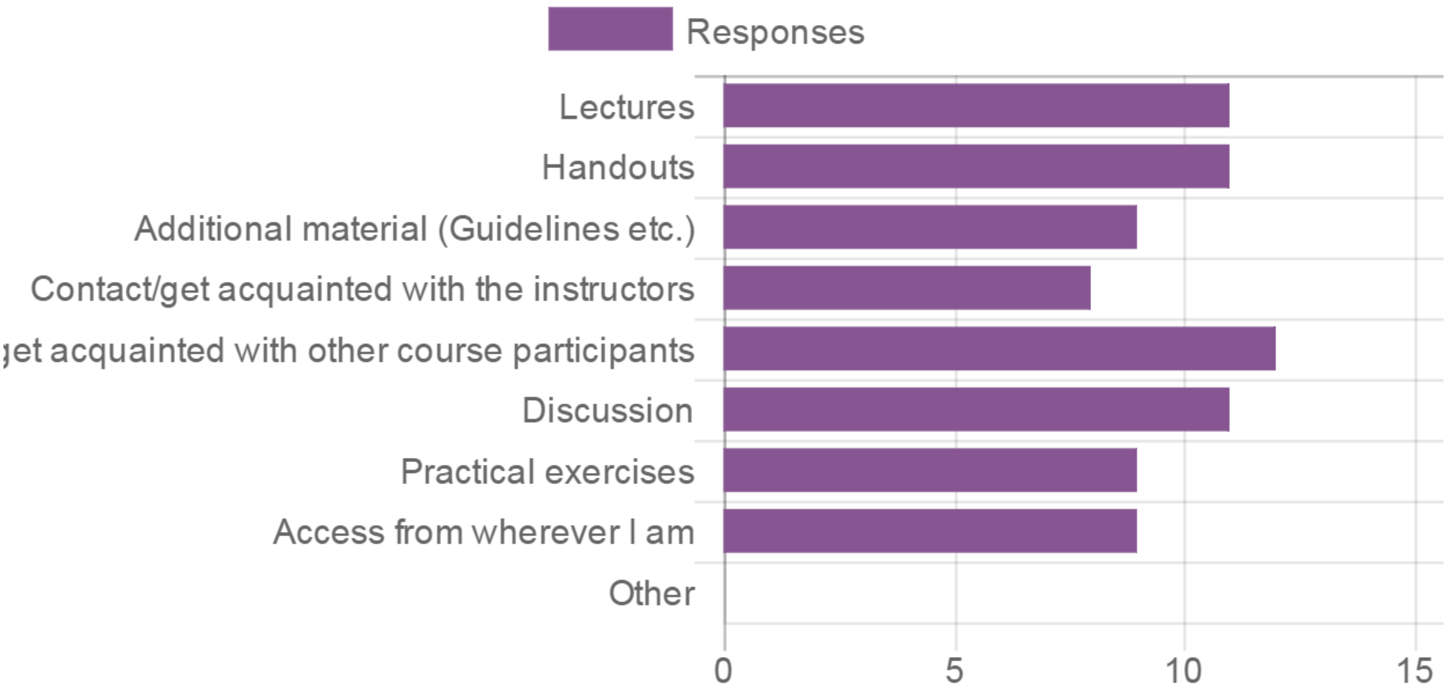

13. Other things that are important to you?

- That the lectures contain references to original articles or books for each statement/allegation in the powerpoint slides.
- I would have preferred if the lectures had had more of a clinical focus rather than being a systematically based on the subject matter.

14. In future courses in specialist education I want MORE…

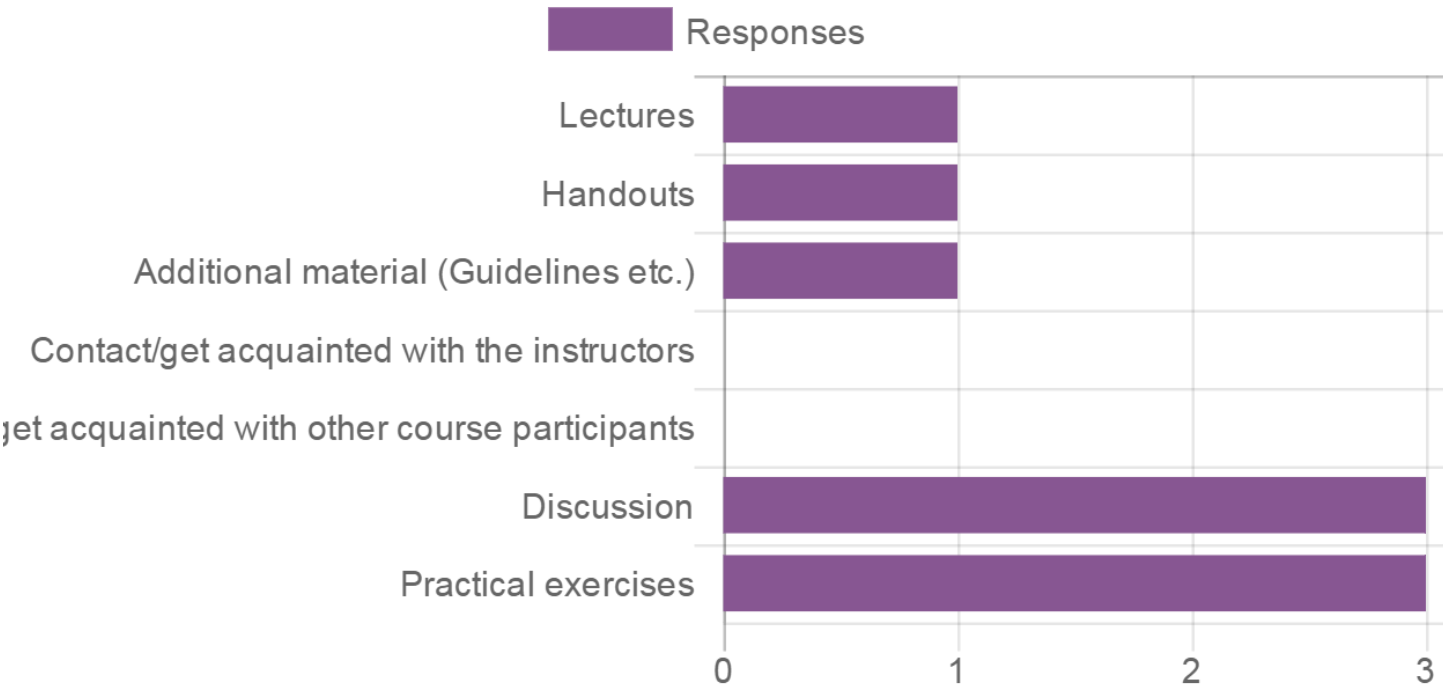

A detailed analysis of the responses reveals that it was the same three of the course participants (25 %) who wanted more discussions (n = 3) and practical exercises (n = 3). One of them additionally wanted more lectures, handouts, and other supplementary materials.

15. Other things that you wish you MORE of?

- I think the course was well laid out, with a little of everything.

16. In future courses in specialist education I want LESS…

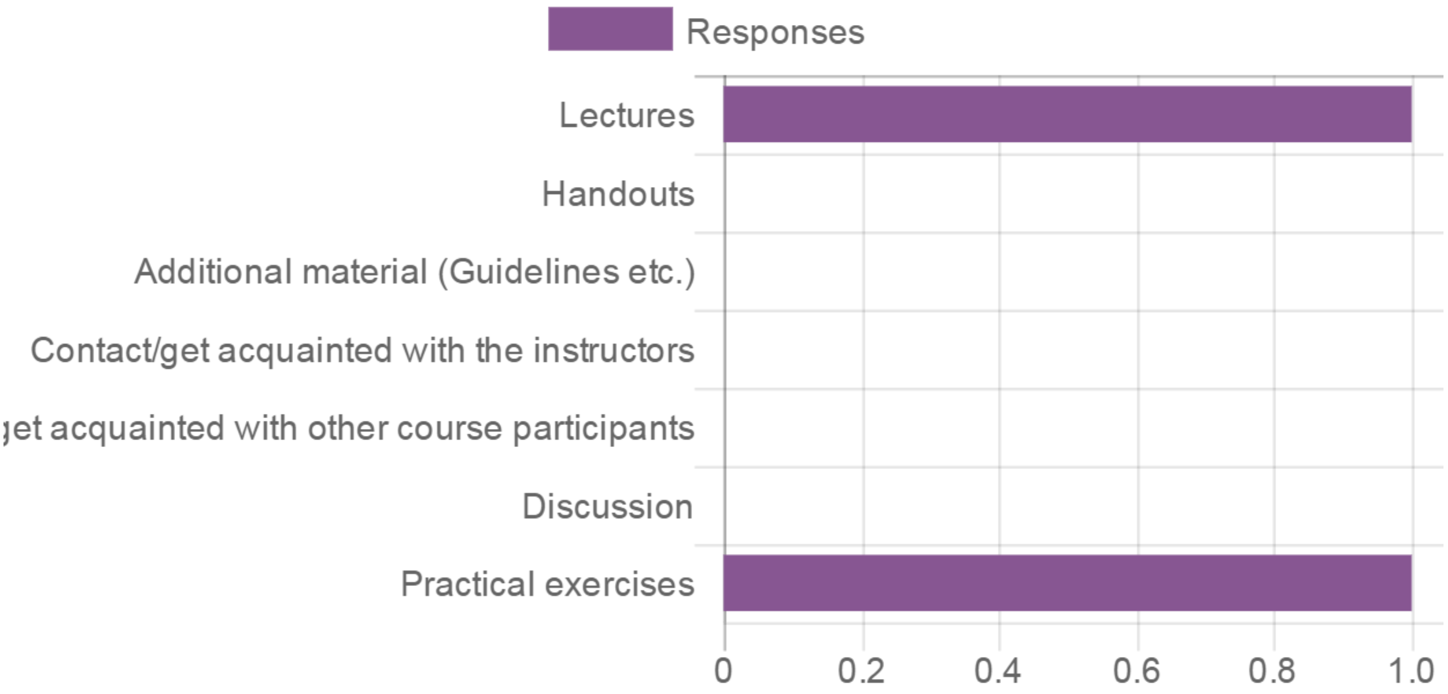

Please note that both bars represent the response from two single (= 1.0) of 12 respondents. A detailed analysis shows that the respondent who wanted fewer lectures had voted for more discussions and practical exercises in question 15. The respondent who wanted fewer practical exercises was otherwise satisfied with the composition of the course.

17. Other things that you want LESS of?

- Not less of *(sic!)*:
  But spent very much time on the course exam (around 20 hours). Some questions were difficult to find the answer in the lectures, and much time passed searching for the right answers in journals/ books etc. The courses Examination could have been shorter, maybe with easier questions.
  By the way, very nice with courses examination via Moodle, which worked well.
- Damaged wall clocks and torn down pictures :)

18. What was particularly good with this course?

- Well organized. Moodle. E-learning.
- Relevant subject matter. Inspiring lecturers.
- Online access to lectures, good lecturers, fairly complete content, comprehensive examination that forces you to go back to the substance.
- Relatively good structure. Exciting with Moodle. Nice experiment with practical exercises, but believe this could be better. Nice dinner together.
- Good teachers, good facilities.
- Group size was perfect.
- Practical exercises in groups of two with a different partner on each day.
- The course was well executed, with fine and thoughtful lectures. Good teachers and helpful and good learning experience for me.
- Scope. Lectures from the whole of Norway.

19. What was especially poor with this course?

- Nothing was particularly poor. See my comment on the course exam.
- Practical exercises were unhelpful when nobody could Oasis. Some exams questions were difficult to find the answer in both lectures and guidelines. Some questions was aimed at how things are done in Bergen, not equally relevant for everybody. The exam was far too large, then you don't manage to really familiarize yourself with the subject matter. I did not find out what was the correct answer in case i had missed out on one choice among several choices. The meeting room was rather confined, and one had to sit on the side of a long table. Those who worked with computers were facing in the wrong direction by 90 degrees.
- Too little time for the practical exercises when the lectures lasted longer than they should.
- Introduction of PET and how cyclotron is built up etc. should come on day 1.
- Some lecturers rattle off a lot of protocols and isotopes incl. research, which is not appropriate for our practice in local hospitals that only conduct the most basic examinations.
- The course exam.
- Too cumbersome course exam.

20. Do you have any more comments? Possible ideas for improvement?

- For someone without experience, the course exam was difficult, and I have spent a LOT of time answering the questions. The positive thing is that we had plenty of time to get it done. Examination questions were mostly relevant and covered the whole course.
- Very good effort and commitment from all of you in Bergen! This will be very nice next time!
  It is very useful to discuss exam questions. Could one have taken each part of the examination on different days?
- Good that the course starts Monday at 9 o'clock, so people can travel to Bergen on the same day, and the course concludes in a reasonable time on Friday so that visitors can come home again.
- Bit tiring to sit at an angle for a whole week ... and no lunch included? - The course was expensive. It was cumbersome having to remember the receipts to get the travel expenses refunded.
- Demanding exam with many questions! Mostly relevant, but too much. Time-consuming in a normal workday.
- I was very pleased with the course. The course exam was too difficult for, but it may be possible because I am an "immigrant" from radiology. I also took the course at the Radium Hospital in 2013 after only one month on nuclear medicine (Worked in radiology again after this), but I think that the course in Bergen was somewhat better.
